# Quantification of muscle recovery methods by near-infrared spectroscopy after heavy exercise in the heat

**DOI:** 10.1186/2046-7648-4-S1-A8

**Published:** 2015-09-14

**Authors:** Sirkka Rissanen, Satu Mänttäri, Juha Oksa

**Affiliations:** 1 Physical Work Capacity, Finnish Institute of Occupational Health, Oulu, Finland

## Introduction

Recovery of muscle function after heavy physical work may last for several hours [1]. This study compared four different recovery modalities to passive recovery during 4 hrs follow-up after heavy exercise period while wearing firefighting protective equipment in a hot environment.

## Methods

Thirteen male firefighters volunteered in the study. They performed an exercise track which contained sledge hammering, going under and over an obstacle, stair walking, hose rolling and walking with and without loads. The exercise lasted for 20 min and the volunteers were asked to perform the exercise as hard as possible but keeping in mind to be able to complete the whole test. They wore firefighting smoke diving protective clothing (ca. 2 clo), helmet, gloves and boots and carried an Air-Pak cylinder in a backpack. Regulator and facemask were not used. Ambient temperature was 35 °C and rh 30 %. Heart rate and rectal temperature were measured during the exercise. Following the exercise, volunteers took off the protective equipment. Recovery process was followed at room temperature for 4 hours. The five recovery modalities were: 1) caffeine ingesting (CI, 6 mg.kg-^1^), 2) forearm stretching (S), 3) forearm cold water (15 °C) immersion (C), 4) forearm cold (15 °C) and warm (37 °C) water immersion (CW) and 5) reference (REF) without any intervention. CI, S, C and CW were applied at 20, 40 and 80 min after the exercise. Recovery follow-up was focused on the right forearm (wrist flexor) and were performed at 0, 30, 60, 120 and 240 min after the exercise and was performed at sitting position. Between the follow-up measurements the participants were free to move. Muscle oxygen consumption (mVO_2_) was measured by near-infrared spectroscopy (NIRS) evaluating the rate of decrease in oxyhaemoglobin in the upper arm. Reoxygenation (ReO_2_) rate was evaluated from the increase in O_2_Hb after the occlusion.

## Results

Mean (SD) rectal temperature rose up to 38.2 (0.1) °C and peak heart rate was 178 (13) b.min^-1 ^during the exercise in the heat. All the four active recovery modalities increased mVO_2 _at 30 and 60 min measurement points compared to REF. At 120 and 240 min post exercise the effects on mVO_2 _were diminished. ReO_2 _rate was decreased by CI at 30 min in comparison to REF. CI and REF were lower (p < 0.05) in comparison to S, C and CW at 60 min. CW demonstrated the most pronounced increase followed by S at 120 min. No differences were observed at 240 in ReO_2_.

## Conclusion

All four active recovery modalities increased muscle VO_2 _and all except CI increased ReO_2 _rate after heavy exercise in the heat in comparison to reference. The most pronounced effect was observed in ReO_2 _rate at 60 and 120 min. The greater increase in mVO_2 _and ReO_2 _rate after these recovery interventions are indicative of increased metabolic rate and probably faster recovery process in the muscle.
